# Host Resistance to Virus Diseases Provides a Key Enabler towards Fast Tracking Gains in Grain Lupin Breeding

**DOI:** 10.3390/plants12132521

**Published:** 2023-06-30

**Authors:** Roger A. C. Jones

**Affiliations:** The UWA Institute of Agriculture, University of Western Australia, 35 Stirling Highway, Perth, WA 6009, Australia; roger.jones@uwa.edu.au

**Keywords:** grain legumes, lupins, virus, disease, yield losses, resistances, breeding, management, research priorities

## Abstract

Four lupin species, *Lupinus angustifolius*, *L. albus*, *L. luteus*, and *L. mutabilis*, are grown as cool-season grain legume crops. Fifteen viruses infect them. Two of these, bean yellow mosaic virus (BYMV) and cucumber mosaic virus (CMV), cause diseases that threaten grain lupin production. Phytosanitary and cultural control measures are mainly used to manage them. However, breeding virus-resistant lupin cultivars provides an additional management approach. The need to develop this approach stimulated a search for virus resistance sources amongst cultivated lupin species and their wild relatives. This review focuses on the progress made in optimizing virus resistance screening procedures, identifying host resistances to BYMV, CMV, and additional viral pathogen alfalfa mosaic virus (AMV), and the inclusion of BYMV and CMV resistance within lupin breeding programs. The resistance types found in different combinations of virus and grain lupin species include localized hypersensitivity, systemic hypersensitivity, extreme resistance, and partial resistance to aphid or seed transmission. These resistances provide a key enabler towards fast tracking gains in grain lupin breeding. Where studied, their inheritance depended upon single dominant genes or was polygenic. Although transgenic virus resistance was incorporated into *L. angustifolius* and *L. luteus* successfully, it proved unstable. Priorities for future research are discussed.

## 1. Introduction

Lupins (*Lupinus* spp.) are grown as annual cool-season grain legume (pulse) crops in all continents apart from Antarctica. The main production areas include countries surrounding the Mediterranean Sea (European, North African, and Middle Eastern), northeast Europe, the Andean region of South America, southern Australia, southern Africa, and southeast USA. Lupin grain is used to feed humans not only directly but also indirectly by providing feed for domestic animals and fishmeal in aquaculture. Depending upon the world region, they are grown as summer crops where the climate is temperate, as winter crops where it is Mediterranean or subtropical, and as wet season, high altitude crops where it is tropical at lower altitudes [1,2,3,4,5,6,7,8]. From 2014 to 2021, the 10 countries that produced the most lupin seed, which are located in Australasia, Europe, South America, and Africa, produced a combined 1.1–1.6 million tonnes of seed annually [9]. The main species cultivated for their grain are narrow-leafed lupin (*L. angustifolius*), white lupin (*L. albus*), yellow lupin (*L. luteus*), and pearl lupin (*L. mutabilis*). Both pearl and white lupins were first domesticated to produce land races in their centres of origin in the Andean region of South America and the Mediterranean region, respectively. The centre of origin of both yellow and narrow-leafed lupin was also in the Mediterranean region. Of these four species, pearl and white lupin were first domesticated >2,500 years ago, whereas narrow-leafed and yellow lupin were domesticated recently in northern Europe (Baltic countries and Germany) [3,5,6,7,10,11]. Lupin land races show evidence of early farmer selection of beneficial traits, such as drought avoidance, reduced vegetative growth, permeable seeds, and greater seed size. However, full domestication of crop lupins requires crossing programs designed to increase other key domestication traits, such as vernalisation insensitivity, low alkaloid levels, early flowering, non-shattering pods, yield stability, and pest and disease resistance, so this did not commence until the early 20th century. This was in Germany in 1928, when the first alkaloid-free lupin plants were isolated [1,2,5,6,7,12]. Although the rough-seeded lupin species sandplain lupin (*L. cosentinii*) was domesticated in the 1970s in Australia, it currently only persists in pastures as feed for domestic animals [1,2,3,5,6,12,13]. Additional lupin species that are recently domesticated, under domestication, or potentially suitable for domestication include three other rough-seeded lupin species, *L. atlanticus*, *L. pilosus*, and *L. digitatus* [5,6,7,14,15], and *L. hispanicus,* which resembles yellow lupin [7]. Lupin species grown as ornamental plants include yellow and pearl lupins, *L. pilosus*, *L. hartwegii*, *L. polyphyllus*, and the interspecies cross *L. polyphyllus × L. arboreus* [5,7]. 

Lupins not only tolerate growing in poor, nitrogen-deficient soils but also contribute nitrogen to the soil, making them ideal for sowing in rotation with crops unable to fix nitrogen. However, they also suffer from diverse abiotic and biotic constraints that limit their productivity [2,3,5,6,12,16,17]. Amongst these constraints, disease is a major contributor, as lupins become infected by a wide range of fungal and viral pathogens that diminish both the yield and the quality of their seeds [18,19,20,21,22]. The magnitude of the disease-induced losses in seed yield and quality that develops varies between different cultivated lupin species, pathogen species and types, climatic differences, and world region [18,19,20,21,22]. This review focuses on virus diseases of grain lupins. It describes the progress made in: (i) developing simple labor-saving procedures that streamline virus resistance screening; (ii) identifying host resistances to BYMV, CMV, and additional viral pathogen alfalfa mosaic virus (AMV) in lupin cultivars, breeding lines, and germplasm accessions, and studying their inheritance; (iii) genetic modification of lupins for virus resistance; and (iv) the inclusion of BYMV and CMV host resistance within lupin breeding programs.

## 2. Viral Pathogens

The major and minor viral pathogens, the disease symptoms they cause, the world regions in which they infect lupins, the different grain lupin species they infect, and their relative importance are listed in Table 1. 

### 2.1. Bean Yellow Mosaic Virus

**Figure 1:**
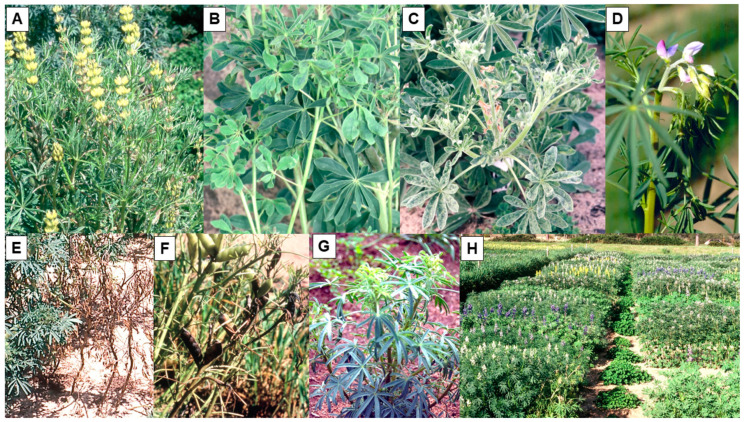
Plants of different lupin (*Lupinus*) species with foliage symptoms caused by infection with bean yellow mosaic virus (BYMV) (**A**–**G**), or being screened for BYMV resistance in the field (**H**). (**A**), Plants of yellow lupin (*L. luteus*) with typical narrow-leaflet symptoms and reduction in leaf size (South Perth 1995). (**B**), Plants of white lupin (*L. albus*) with typical leaf symptoms of mosaic and deformation (front) and unaffected plants with larger dark green leaves (behind) (South Perth 1997). (**C**), Plant of sandplain lupin (*L. costentinii*) with typical leaf symptoms of severe mosaic and deformation, and reduction in size (front) with unaffected plant (behind) (South Perth 1989). (**D**), Plant of narrow-leafed lupin (*L. angustifolius*) with typical initial early necrotic strain symptom consisting of shoot tip bending over (‘shepherds crook’) (South Perth 1989). (**E**), Three plants of narrow-leafed lupin killed by early necrotic strain infection (right), and healthy plant (left) (South Perth 1992). (**F**), Plant of narrow-leafed lupin with black pod syndrome caused by late necrotic strain infection (centre), and healthy plant with normal-looking pods (top left) (South Perth 1995). (**G**), Plant of narrow-leafed lupin with typical chlorosis and downcurling of leaflets in apical leaves caused by recent infection with the non-necrotic strain (Avondale 1995). (**H**), Single row plots of cultivars, breeding lines, and germplasm accessions of different lupin species undergoing BYMV resistance screening (South Perth 1993). Note the BYMV-infected clover transplants positioned at both ends of each row to provide a uniform infection source for naturally occurring aphid vectors to spread the virus.

The most important, widespread, and damaging viral pathogen of grain lupins is bean yellow mosaic virus (BYMV) [18]. Although BYMV causes a mild disease in pearl lupin, a damaging disease develops in the other four cultivated lupin species (Table 1). Its principal foliage symptoms vary between lupin species: pearl lupin—mild mosaic and slight plant stunting; yellow lupin—narrowing of leaflets, vein mosaic, bunchy growth, and plant dwarfing; and both white and sandplain lupin—severe mosaic, necrotic spotting and deformation of leaves, and plant stunting (Figure 1A–C) [18]. In narrow-leafed lupin, BYMV symptom development depends upon the virus strain present (necrotic or non-necrotic) and the growth stage when infection occurs. Early infection with the necrotic strain causes bending over of the shoot tip, necrotic stem streaking, and plant death (Figure 1D,E), whereas late infection of mature plants remains restricted to one or some branches, which develop black pod syndrome (BPS) and/or systemic necrosis (Figure 1F) [18,52]. In contrast, the necrotic phenotype is lacking when plants become infected by the non-necrotic strain, which causes mosaic and stunting symptoms (Figure 1G) [53,54]. The earliest reports of virus symptoms resembling those caused by BYMV were in yellow lupin in Germany in 1929, in Argentina in white lupin in 1932, and in narrow-leafed lupin in New Zealand in 1934. During the period from 1938 to 1960, typical BYMV symptoms were reported under different names in plants of these three lupin species in Europe, Australasia, North America, and Southern Africa. They were also reported in pearl lupin in Australia, New Zealand, and South Africa, and in sandplain lupin in Australia [18]. Because it occurs worldwide [55,56,57], BYMV infection poses a serious threat to the lupin crop wherever it is grown in the world. It infects many species of flowering plants (both monocots and dicots) and causes damaging diseases in legume species [23,24,55,57]. It is vectored non-persistently by >50 aphid species, including *Myzus persicae*, *Aphis craccivora*, *A. fabae*, *Acyrthosiphon kondoi*, *Acyrthosiphon pisum*, and *Macrosiphum euphorbiae* [18,23,24,58]. It is readily seed-borne in yellow and white lupin, and sowing their infected seed stocks creates primary infection foci from which aphid vectors spread the virus within the crop. In contrast, seed transmission has never been found in narrow-leafed, pearl, or sandplain lupin. Therefore, with them, lupin crop infection depends solely on aphid vectors bringing in BYMV from infected alternative hosts growing nearby, such as legume weeds, pasture plants, and crops [18,59]. Weather conditions that promote aphid build-up both before and during the growing season (especially rainfall and warm temperatures) favor its spread within lupin crops [59,60].

Phytosanitary (sow healthy seeds, isolate from external virus sources, and sow perimeter non-host barrier crop), cultural (sow early maturing cultivars, deter aphid landings with stubble groundcover, and promote early canopy closure), and chemical (apply insecticide to suppress aphid vectors in adjacent legume pasture) control measures are available for managing BYMV in lupin crops [18,19,25,59,61]. However, host resistance offers an alternative approach towards BYMV management [18]. In the 1950s in southeast USA, selections of yellow lupin and 31 other lupin species were screened for BYMV resistance [26,27,28]. None was found in any cultivated lupin species, but six perennial lupin species had extreme BYMV resistance (immunity). Unfortunately, all attempts to transfer this resistance to yellow, white, and narrow-leafed lupin were unsuccessful. Similarly, from 1970 to 1980 in Europe, screening for BYMV resistance found none in yellow lupin breeding selections in Poland [29] or in yellow or white lupin cultivars in Hungary [30]. In Byelorussia, however, screening of 21 lupin species for resistance to BYMV found some ‘resistant’ lines (= no symptoms developed, so some might have been tolerant) amongst several wild lupin species and one ‘resistant’ pearl lupin line (K2153) [31]. In Russian studies, partial resistance to BYMV infection by aphids was reported in several yellow and white lupin breeding lines [32]. In Ukraine, similar partial resistance was found in yellow lupin cv. Motiv [33]. In addition, when resistance to BYMV seed transmission was studied in Russia, yellow lupin breeding lines with ‘intrinsic’ BYMV seed transmission rates as low as 3% were identified despite 30% being the typical seed transmission rate [33]. Moreover, when 102 breeding lines and other populations of yellow lupin were tested for possible BYMV resistance in Germany, 21 of them had quantitatively inherited partial resistance, which was linked to reduced seed transmission [34]. Similar resistance to BYMV seed transmission was reported in yellow lupin in Poland [35]. Within Eastern European yellow and white lupin breeding programs, therefore, large-scale BYMV resistance screening focused on partial resistance to infection by aphids and resistance to seed transmission [33,36,37]. This was achieved by field exposure in the presence of ‘spreader rows’ sown with BYMV-infected lupin seed or spray gun inoculation [33].

**Figure 2:**
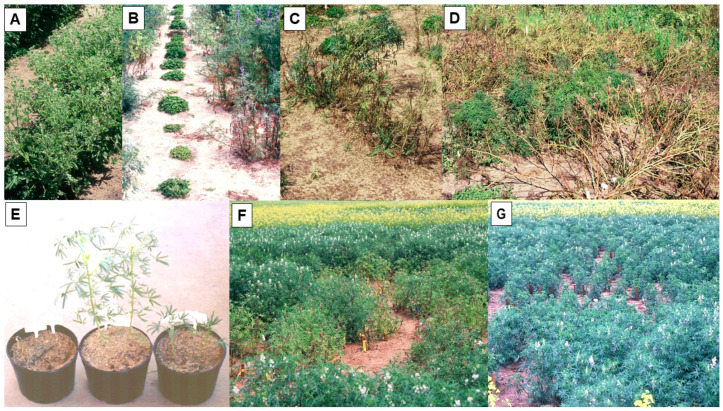
Plants of the rough-seeded lupin species sandplain lupin *(Lupinus costentinii*) or narrow-leafed lupin (*L. angustifolius*) being screened for resistance to the necrotic strain of bean yellow mosaic virus in the field (**A**–**D**), and plants of narrow-leafed lupin infected with its necrotic or non-necrotic strains being evaluated for symptom expression in the glasshouse (**E**) or their patterns of spread in the field (**F**,**G**). (**A**), Single row plot of sandplain lupin with all plants infected showing symptoms of severe mosaic and leaf deformation, reduction in leaf size, and stunting (South Perth 1995). (**B**), Plants of narrow-leafed lupin closest to infected subterranean clover transplants showing systemic necrotic symptoms after being the first ones to become infected (South Perth 1992). (**C**), Row of infected narrow-leafed lupin plants showing systemic necrotic symptoms (South Perth 1992). (**D**), Row of narrow-leafed lupin germplasm accession P26697 in which plants show systemic mosaic and leaf deformation symptoms without necrosis (centre), and rows of other accessions killed by infection (front and on top left behind) (South Perth 1995). (**E**), Plants of narrow-leafed lupin cv. Danja (2/pot) left uninoculated (centre), and aphid-inoculated with the necrotic strain (left) or the non-necrotic strain (right). Infected plants both killed (necrotic strain) or severely stunted without any necrosis (non-necrotic strain) (South Perth 1994). (**F**,**G**), Plots of narrow-leafed lupin cv. Gungurru within which necrotic or non-necrotic strains were being spread from centrally placed infection foci (infected clover transplants) by naturally occurring aphids (Avondale 1999). (**F**), Slow, localized spread of the necrotic strain killing plants in the plot central region. (**G**), Faster spread of the non-necrotic strain causing more widespread infection of plants that became stunted without developing any necrosis. Stakes mark individual infected plants.

In Australia, annual routine field screening for resistance to the necrotic BYMV strain commenced for the Australian national lupin breeding program in 1989 [38,39]. Single row plots of lupin test lines were exposed to uniform BYMV inoculum pressure by placing BYMV-infected subterranean clover transplants at each of their ends and allowing naturally occurring aphid vectors to spread the virus along the rows (Figure 1H). Over the years, this annual BYMV resistance screening included not only very large numbers of narrow-leafed lupin germplasm accessions, breeding lines, and cultivars, but also smaller numbers of yellow, white, pearl, and rough-seeded lupins (Figure 2A). Although no extreme BYMV resistance was ever found in any lupin species, two different types of BYMV resistance were detected in narrow-leafed lupin: systemic hypersensitive resistance (SHR) and the partial resistance to BYMV transmission by aphids found previously in yellow and white lupin in Europe (see previous paragraph) [38,39]. SHR (i.e., the typical systemic necrosis and plant death syndrome that results from early BYMV infection) was exhibited by all of the numerous narrow-leafed lupin lines evaluated, apart from accession P26697 and lupin breeding line 90L423-07-13, both of which always developed a ‘non-necrotic’ phenotype (Figure 2B–D) [39,54,59]. SHR is called a resistance reaction because it is controlled by single resistance genes and limits virus spread in the field [62,63]. When a diverse range of necrotic and non-necrotic strain isolates were aphid-inoculated to plants of narrow-leafed lupin cultivars Danja and/or Merrit and of 90L423-07-13 and/or P26697, all of the necrotic strain isolates (but none of the non-necrotic strain isolates) elicited SHR phenotypes when inoculated to Danja or Merrit (Figure 2E) [54]. In contrast, only two of the necrotic strain isolates (neither of which came from lupin) and none of the non-necrotic strain isolates elicited SHR phenotypes when inoculated to 90L423-07-13 or P26697. This suggested the presence of two putative strain-specific, independently inherited SHR genes and four BYMV strain groups (= pathotypes). Strain group 1 contained the two isolates that elicited necrotic phenotypes with both putative SHR genes. Strain group 2 contained the isolates that elicited the putative gene in the two cultivars but not the putative gene in 90L423-07-13 and P26697. Strain group 3 is made up of hypothetical isolates that only elicit the putative gene in 90L423-07-13 and P26697. Strain group 4 contained isolates that elicited neither putative gene, and therefore always caused non-necrotic phenotypes (= the non-necrotic strain [54]. 

Proof that the putative SHR gene present in Danja and Merrit exists was obtained following inoculation of a strain group 2 isolate to F2 progeny plants of six different crosses [62]. A 3:1 ratio for necrotic:non-necrotic phenotypes was obtained with the crosses 90L423-07-13 × Danja, 90L423-07-13 × Merrit, P26697 × Danja, and P26697 × Merrit, but entirely non-necrotic or 99% necrotic phenotypes were obtained with 90L423-07-13 × P26697 or Danja × Merrit, respectively. This single, independently inherited, dominant SHR gene was named *Nbm-1* [62]. Moreover, evidence was obtained that independently segregating modifier genes present in the genetic background altered necrotic phenotype expression elicited by *Nbm-1*. This was because in F2 progeny plants derived from crosses between parents with and without *Nbm-1*, the delay between inoculation and the plant being killed varied markedly from plant to plant [62]. This delay was most evident when P26697 was a parent. Proving that the second putative SHR gene exists would require inoculation of a subgroup 3 isolate to progeny plants of similar crosses. Because all narrow-leafed lupin genotypes apart from 90L423-07-13 and P26697 developed SHR when infected with necrotic BYMV strain isolates from lupins, there is no need for active *Nbm-1* gene incorporation into new narrow-leafed cultivars. However, the inadvertent selection of new cultivars that behave like 90L423-07-13 and P26697 should be avoided when advanced narrow-leafed lupin breeding lines are screened for BYMV resistance in the field. Furthermore, a search for resistance to the non-necrotic BYMV strain would be worthwhile as, by spreading faster, it causes greater yield losses [63]. Both *Nbm-1* and the second putative SHR gene were absent from other cultivated lupin species as, during routine BYMV resistance screening, the rapid necrosis followed by death syndrome never developed in any of them [54]. The suspected quantitatively inherited partial resistance trait in narrow-leafed lupin was characterised by the need for inoculation by many more viruliferous aphids to establish necrotic phenotype infection successfully and was unrelated to aphid susceptibility, flowering date, or alkaloid content [39]. Breeding line 84A086-5-20-31 had outstanding partial resistance of this type both under routine BYMV field screening conditions and in larger-scale field evaluations. Therefore, it seems likely to be a suitable parent for crosses focused on breeding narrow-leafed lupin cultivars destined for BYMV-prone regions [39,59,64].

The question arises as to how the presence of the SHR gene *Nbm-1* would be beneficial to narrow-leafed lupin crops growing in the field despite the rapid killing of plants infected early by the necrotic strain, which then produce no seeds. The answer is that instead of intervening to prevent virus spread at the level of individual plants, SHR does this at the plant population level. Thus, the killing of plants infected early by the necrotic BYMV strain prevents them from becoming a virus source for further spread by aphid vectors (Figure 2F) [65,66,67,68]. In contrast, because the non-necrotic strain breaks this resistance by overcoming *Nbm-1*, lupin plants infected with it remain alive throughout the life of the crop, acting as sources for further virus acquisition and spread by naturally occurring aphid vectors, which results in many more infected plants (Figure 2G) [66,67]. The greater yield losses caused when the non-necrotic strain infected more plants was demonstrated clearly in large-scale field experiments in which both strains were introduced into narrow-leafed lupin plots and allowed to spread by naturally occurring aphids [63]. In contrast, when both strains infected subterranean clover plants, the necrotic strain outcompeted the non-necrotic strain. This explains why there are always more primary infection foci of the former than the latter when BYMV spreads from BYMV-infected subterranean clover pastures into narrow-leafed lupin crops [53,54]. 

When late infection with the necrotic BYMV strain occurred in narrow-leafed plants in the field, cv. Mandellup was ranked as more ‘BPS-susceptible’ than cv. Jenabillup [69]. However, sap inoculation of the necrotic BYMV strain to plants at different growth stages failed to confirm this because, although its development was slower in Jenabillup than in Mandellup, the BPS symptoms that formed later were as severe as those in Mandellup [52]. Despite this finding, the slower BPS development in Jenabillup might still be a trait of interest for future breeding for BPS resistance in narrow-leafed lupin. Therefore, further studies on BPS are warranted, including obtaining an understanding of its genetic basis (likely polygenic) and the mechanism responsible for it (e.g., mature plant resistance or partial resistance to systemic infection via the phloem) [52].

The possibility of using genetic engineering to introduce BYMV resistance to narrow-leafed and yellow lupins was also investigated [70,71]. Different protease (NIa) gene constructs derived from BYMV were introduced to both lupin species. However, when later generation transgenic progeny plants were inoculated with BYMV, only partial resistance (slow systemic movement) was found. This was restricted to some yellow lupin plants but was absent from any narrow-leafed lupin plants [72,73]. In addition, a synthetic ‘hairpin’ construct derived from the replicase (NIb) gene of BYMV was introduced to plants of narrow-leafed lupin cv. Wonga [74]. When the progeny plants of forty-five lines with this construct were inoculated with BYMV, seven of them had extreme resistance. However, when later generation progeny plants were tested, the resistance derived from the NIb gene construct had become silenced [40].

### 2.2. Cucumber Mosaic Virus 

**Figure 3:**
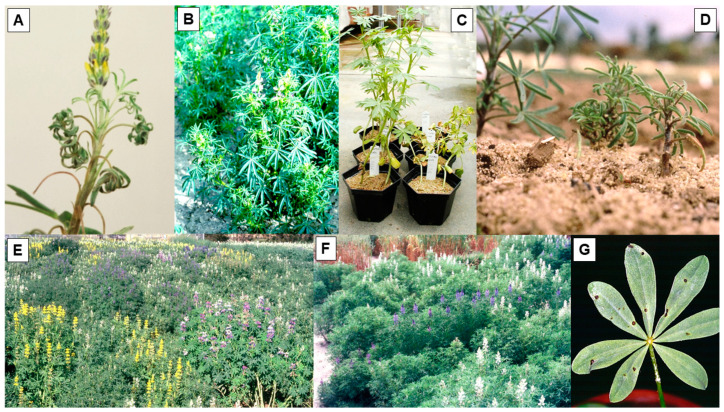
Plants of different lupin (*Lupinus*) species with foliage symptoms caused by cucumber mosaic virus infection (**A**–**G**). (**A**), Plant of yellow lupin (*L. luteus*) showing typical leaflet symptoms of downcurling and reduction in size (South Perth 1994). (**B**), Plant of narrow-leafed lupin (*L. angustifolius*) with typical leaflet symptoms of chlorosis, leaflet downcurling and reduction in size, and plant stunting (front) with healthy plants (behind) (Wongan Hills 1995). (**C**), Three plants of pearl lupin (*L. mutabilis*) inoculated with infective sap showing typical leaflet symptoms of mosaic, chlorosis, deformation and reduction in size, and plant stunting (right), and three uninoculated control plants (left) (South Perth 2003). (**D**), Two plants of a narrow-leafed lupin germplasm accession with seed-borne infection showing typical symptoms of leaflet downcurling and reduction in size and plant stunting (right), and plant grown from healthy seed (left) (South Perth 1986). (**E**), Single row plots of cultivars, breeding lines, and germplasm accessions of different lupin species undergoing resistance screening, and separated on each of their sides by single spreader row plots sown with infected seed of a narrow-leafed lupin cultivar with a high seed transmission rate to provide a uniform virus infection source for naturally occurring aphid vectors to spread the virus (South Perth 1993). (**F**), Screening of narrow-leafed lupin breeding lines and germplasm accessions showing irregular plant growth arising from gradual spread of infection within and between alternating spreader rows and test rows (South Perth 1992). (**G**), Necrotic local lesions in leaf of yellow lupin cv. Motiv inoculated with infective sap (South Perth 1994).

The second most widespread and damaging viral pathogen of grain lupins is cucumber mosaic virus (CMV), which causes a damaging disease in yellow, narrow-leafed, and pearl lupins [18]. *L. hispanicus*, a close relative of yellow lupin, is also a CMV host [41], but white lupin and the rough-seeded lupin species are all non-hosts (Table 1) [18,41]. Its principal foliage symptoms following current season infection of yellow, narrow-leafed, and pearl lupins are mosaic, leaflet downcurling, leaf chlorosis, diminished leaf size, and plant stunting (Figure 3A–C) [18,41,42,43]. The earliest reports of virus symptoms resembling those caused by CMV were in yellow and narrow-leafed lupins in Germany in 1934, in Holland in 1936, and in New Zealand in 1939. CMV was identified as the causal agent in Germany in 1935. During the period from 1950 to 1970, CMV was reported infecting one or both of these two lupin species in Poland, Russia, the USA, and South Africa, and by 1981 in Australia [18]. In 1986, it was reported infecting pearl lupin in Germany [44]. Because it occurs worldwide [56,57,75], CMV infection poses a serious threat to narrow-leafed, yellow, and pearl lupin crops wherever they are grown in the world. Like BYMV (see Section 2.1 above), it also infects many other species of flowering plants (both monocots and dicots), causing damaging diseases in legume species [45,56,57,75]. It is vectored non-persistently by >80 aphid species, including *M. persicae*, *A. craccivora*, *A. fabae*, *Acyrthosiphon kondoi*, *Acyrthosiphon pisum*, and *Macrosiphum euphorbiae* [18,23,24,58,75]. CMV is readily seed-borne in narrow-leafed and yellow lupin, and sowing their infected seed stocks creates primary infection foci from which aphid vectors spread the virus within the lupin crop (Figure 3D) [18,59,76]. In contrast, no CMV seed transmission has been found in pearl lupin [41,44]. When seed-infected lupin plants are absent within crops of CMV-susceptible lupin species, as with BYMV in lupins, crop infection depends solely on aphid vectors bringing it from infected alternative hosts growing nearby, such as legume weeds, pasture plants, and crops [18,59]. As with BYMV, climatic conditions that promote aphid build-up both before and during the growing season (particularly warm temperatures and rainfall) favour CMV spread within lupin crops [59,77].

The control measures used to manage CMV in lupins are phytosanitary (sow seed stocks with below threshold % infection appropriate for the CMV risk zone and isolate from external virus sources), cultural (sow early maturing cultivars, generate high plant densities within rows to shade out seed infected plants, deter aphid landings with stubble groundcover, and promote early canopy closure), and chemical (apply selective herbicide to remove weed hosts of aphid vectors) [19,59,61,76]. However, CMV control is also possible via host resistance [18]. No CMV resistance searches involving different cultivated lupin species have been reported outside Australia, but in 1987, the Australian national lupin breeding program started annual routine field screening for CMV resistance [43]. This same CMV resistance screening procedure developed in 1987 is still being employed annually by the lupin breeding program 36 years later (i.e., in 2023). Over the years, it has focused mainly on narrow-leafed lupin germplasm accessions, breeding lines, and cultivars, but smaller numbers of yellow, white, pearl, and rough-seeded lupin species and *L. hispanicus* accessions have sometimes been included (Figure 3E). This resistance screening approach achieves a uniform CMV inoculum source by sowing spreader rows of a narrow-leafed lupin cultivar with a high rate of seed infection on either side of every single row test plot. Naturally occurring aphid vectors spread CMV to lupin plants in the test plots (Figure 3F). Seed is harvested from every test plot and germinated, and the seedlings are tested for CMV to determine the % seed transmission rate. Three different types of CMV resistance were detected in these test plots: resistance to seed transmission in yellow and narrow-leafed lupin, strain-specific localized hypersensitive resistance (LHR) in yellow lupin and *L. hispanicus*, and extreme resistance in pearl lupin [41,43,46,47]. In addition, narrow-leafed lupin genotypes differed in sensitivity to infection as current-season CMV symptoms ranged from mild to severe in the different genotypes [43]. Whether partial resistance to infection by aphids resembling that found previously with BYMV in yellow and white lupin in Europe (see Section 2.1 above) was present in narrow-leafed lupin could not be established. This was due to the intense CMV inoculum pressure from vector aphids which moved it from the spreader rows and infected all of the plants. Nonetheless, in narrow-leafed lupin breeding trials in which CMV infection was spreading naturally, the amount of spread often varied widely between the different genotypes present. The presence of partial resistance to CMV infection by aphids in some genotypes would seem a possible cause of such differences, as would unevenly distributed original CMV inoculum sources, e.g., amongst the seeds of the different lupin genotypes sown. To obtain proof of partial resistance to CMV infection by aphids in narrow-leafed lupin genotypes, field experiments involving large, replicated plots sown with healthy seeds of different genotypes combined with a smaller but still uniformly distributed CMV inoculum source would be needed [59].

When seed samples harvested from each of the Australian national lupin program’s annual routine field screening test plots of narrow-leafed lupin were tested for CMV seed transmission to seedlings, differences in CMV seed transmission rates were highly significant and stable between individual germplasm accessions, breeding lines, and cultivars [43]. For each genotype, their intrinsic CMV seed transmission rates were classed as (% seed transmission to seedlings in parentheses): ‘very susceptible’ (35–75%), ‘susceptible’ (20–35%), ‘moderately susceptible’ (6–20%), and ‘moderately resistant’ (1–6%). However, none were ever classed as ‘highly resistant’ (<1%) nor ‘immune’ (0%) to CMV seed transmission. An analysis of seed samples from the progeny plants of crosses between genotypes with low and high seed transmission rates provided evidence of transgressive segregation for resistance to seed transmission. Therefore, seed transmission differences between genotypes were inherited quantitatively and under polygenic control, and were unrelated to alkaloid content or flowering time [43]. Based on these findings, in 1994, the lupin breeding program started breeding for ‘moderate resistance’ to seed transmission [78]. This breeding effort not only made possible the release of new narrow-leafed lupin cultivars with very low intrinsic CMV seed transmission rates [48,59,76], but also the removal of all genotypes within the ‘susceptible’ or ‘very susceptible’ classes. Within the routine field screening test plots of yellow lupin, the CMV seed transmission rates to seedlings found in seed samples harvested from different genotypes ranged from of 0.2 to 16% [41]. As the seed transmission rate differences between genotypes were stable across the years, this showed that partial resistance to CMV seed transmission was present, resembling that found in narrow-leafed lupin.

**Figure 4:**
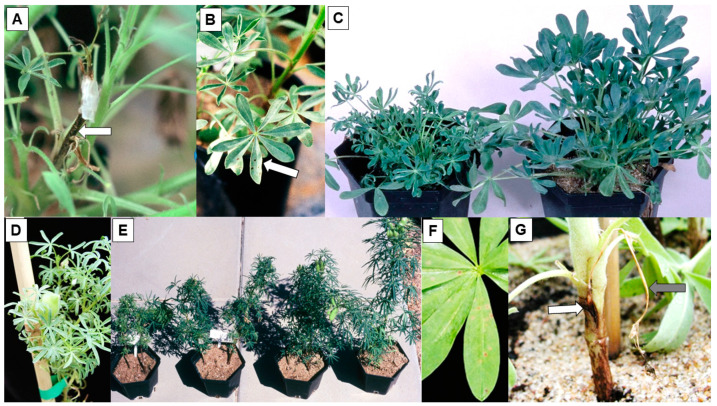
Plants of different lupin (*Lupinus*) species with foliage symptoms caused by infection with cucumber mosaic virus (CMV, (**A**–**C**)), alfalfa mosaic virus (AMV, (**D**,**F**,**G**)), or both viruses (**E**). (**A**), Plant of yellow lupin cv. Popiel graft inoculated with a CMV-infected scion showing symptoms of localized stem necrosis immediately below the graft union (localized hypersensitive resistance, LHR) (white arrow) (South Perth 1994). (**B**), Plant of *L. hispanicus* accession P26858 showing large necrotic local lesions (LHR) in leaves inoculated with CMV-infective sap (white arrow) (South Perth 1993). (**C**), Plants of *L. hispanicus* accessions P26853 (left) and P26858 (right) inoculated with CMV-infective sap showing a susceptible phenotype of systemic mosaic, leaflet downcurling and reduction in leaf size, and plant stunting (P26853), and an LHR phenotype consisting of necrotic local lesions without systemic invasion (P26858) (South Perth 1993). (**D**), Plant of narrow-leafed lupin cv. Tanjil inoculated with AMV-infective sap showing systemic leaflet symptoms of pallor, mild mosaic, leaflet downcurling, and reduction in size (South Perth 2006). (**E**), Plants of narrow-leafed lupin cv. Gungurru (2/pot) inoculated with infective sap of both viruses (left), CMV alone (centre left), AMV alone (centre right), or left uninoculated (right): severe stunting (both viruses), moderate stunting (CMV), mild stunting (AMV), and without stunting (healthy) (South Perth 1996). (**F**), Brown necrotic local lesions (LHR) in leaf of pearl lupin inoculated with AMV-infective sap (South Perth 2006). (**G**), Plant of rough-seeded lupin species *L. digitatus* graft inoculated with an AMV-infected scion (now dead, grey arrow) showing symptoms of localized stem necrosis immediately below the graft union (LHR) (white arrow) (South Perth 2006).

Amongst the yellow and pearl lupin, and the *L. hispanicus,* genotypes included within the routine CMV field screening test plots, some genotypes of each species remained uninfected [41,47]. Sap and graft inoculation of plants of the uninfected yellow lupin genotypes found LHR in all plants of three cultivars (Popiel, Teo, and Motiv) and one breeding line (WTD1191) (Figure 3G and Figure 4A). These genotypes all came from Poland or Byelorussia. Moreover, this LHR was effective against 8/9 CMV isolates, which included the five isolates originally from lupins. Therefore, it had broad specificity, but was still strain-specific. When sap inoculated with CMV, F2 progeny plants of crosses between susceptible and resistant parents gave a 3:1 (necrotic:non-necrotic) ratio. Therefore, this LHR phenotype was controlled by a single dominant gene to which the name *Ncm-1* was given. The Polish and Byelorussian lupin breeders were unaware that it was present in their lupin breeding material. Because of its broad specificity, the active incorporation of gene *Ncm-1* into yellow lupin breeding programs was recommended. Accession P26815 from Portugal developed an SHR phenotype following graft inoculation, so it did not carry gene *Ncm-1* [41]. Three *L. hispanicus* genotypes that remained uninfected during routine field screening for CMV resistance were sap inoculated with five CMV isolates. In accession P26858, an LHR phenotypic response was obtained with 4/5 isolates, so its broad strain specificity resembled that of the yellow lupin genotypes carrying gene *Ncm-1* (Figure 4B). In contrast, accessions P26859 and P26853 only developed an LHR phenotype following inoculation with 1/5 CMV isolates, developing susceptible phenotypes with the four others. They therefore lacked the broad strain specificity LHR present in P26858 (Figure 4C). Therefore, two distinct LHR specificities were present in *L. hispanicus* [41]. 

When plants of the single pearl lupin genotype (accession P26956) that remained uninfected during routine field screening for CMV resistance were sap inoculated with six CMV isolates, no infection was ever established in them [47]. In contrast, simultaneous sap inoculations to plants of susceptible control pearl lupin genotype P26961 always resulted in a systemic susceptible phenotype. Graft inoculation of CMV to P26956 plants caused localized necrosis restricted to the stem region immediately below the graft union without further systemic invasion. When no LHR or other infection occurs in sap-inoculated leaves, this graft inoculation phenotype is typical of extreme resistance [79]. P26956 therefore seems likely to carry an extreme resistance gene for CMV resistance. If so, that would be of considerable interest for breeding new pearl lupin cultivars with CMV resistance [47].

Use of genetic engineering approaches to introduce CMV resistance into lupin was studied first using plants of tobacco as a model system [80]. When transgenic tobacco (*Nicotiana tabacum*) plants with CMV coat protein (CP) or defective replicase (DR) gene constructs were challenged by inoculation with CMV isolates from lupin, the transgenic plants remained uninfected, systemic spread was delayed, there was symptom remission, or some plants were fully susceptible. Later research investigated the performance of *N. benthamiana* plants transformed using CP, DR, or viral movement protein gene constructs of a CMV isolate from lupin. Some of the transformed *N. benthamiana* plants with CP, DR, or viral movement protein gene constructs had extreme resistance to CMV or delayed symptom expression [81,82,83]. Although narrow-leafed lupin plants were transformed successfully using these gene constructs [83], the transgenic CMV resistance found seemed unstable in plants of their later generations. 

### 2.3. Alfalfa Mosaic Virus 

Another widespread virus of less importance to global lupin production, but to which host resistance has been found in lupins, is alfalfa mosaic virus (AMV). The first reports of it infecting cultivated lupin came from Europe, where it was found infecting ornamental Russell hybrid lupin (*L. polyphyllus* × *L. arboreus*) in the UK in 1960, yellow lupin in Poland in 1977, both yellow and white lupins in Germany in 1981, and narrow-leafed lupin in Australia in the 1980s [18]. Its principal foliage symptoms following current season infection of cultivated lupins include mosaic, leaflet downcurling, diminished leaf size, and plant stunting [18,49]. It occurs worldwide and has a wide host range, thereby infecting many other species of flowering plants, including crop, pasture, and weed legume species [56,57,84]. It is transmitted non-persistently by >20 aphid species, which include *M. persicae*, *A. craccivora*, *A. fabae*, *Acythosiphon kondoi*, *Acyrthosiphon pisum*, *Therioaphis trifolii*, and *Macropsiphum euphorbiae* [23,24,84,85]. AMV-infected legume pastures are important sources for its spread by naturally occurring aphid vectors to adjacent lupin crops [49]. AMV seed transmission to seedlings (0.8%) was found in narrow-leafed lupin, so sowing infected lupin seed provides an alternative primary infection source for its spread within lupin crops [49].

In European studies, when AMV was inoculated to white lupin plants, it caused an LHR phenotype consisting of necrotic spots in inoculated leaves without systemic invasion [50,51]. In Australian studies, sap or graft inoculation of yellow lupin, narrow-leafed lupin, and *L. hispanicus* plants with AMV always elicited susceptible phenotypes [49]. In yellow lupin, this phenotype was asymptomatic, but occasional necrotic line patterns in leaves and mild plant stunting developed in *L. hispanicus*. In narrow-leafed lupin, foliage symptoms were mild mosaic, leaflet downcurling, and plant stunting, representing a milder version of the CMV symptoms that appear in this lupin species (see Section 2.2 above) (Figure 4D). However, more severe symptoms appeared in mixed infections with AMV and CMV than when either virus was present alone (Figure 4E) [49]. In contrast, when plants of white lupin and two rough lupin species (sandplain lupin and *L. pilosus*) were sap inoculated with AMV, they all remained uninfected, whereas plants of pearl lupin and two other rough-seeded lupin species (*L. atlanticus* and *L. digitatus*) all developed LHR phenotypes in inoculated leaves (Figure 4F). Following graft inoculation with AMV, all plants of pearl lupin and *L. digitatus*, and some plants of white lupin, developed localized necrosis directly below the graft union (Figure 4G), and a single plant (1/6) of *L. pilosus* developed SHR. The others remained uninfected. All sandplain lupin plants remained uninfected. In this study, however, only one AMV isolate was used, and only one to two genotypes each of white lupin, pearl lupin, *L. hispanicus*, and the four rough-seeded lupins were inoculated [49]. As mentioned in the previous paragraph, white lupin had been reported as an AMV host in Europe previously. Therefore, more research is required to establish whether the LHR, SHR, and extreme AMV resistance phenotypes found in these lupin species represent host resistances of potential value for future AMV resistance breeding in lupins, and whether any useful AMV resistance is present in yellow or narrow-leafed lupin. 

### 2.4. Other Viruses

Twelve other viruses have been found infecting one or more lupin crop species in different parts of the world, but all are currently of minor importance (Table 1). None of these viruses have been studied to establish whether any of the cultivated lupin species they infect have resistance to them.

### 2.5. Molecular Approaches 

The phenotype-based virus resistance screening procedures described above in this review have proven very effective in identifying different categories of host resistance to BYMV and CMV in grain lupin species and in incorporating them into new cultivars. However, although molecular approaches enabling the identification of quantitative trait loci (QTLs) and the development of molecular markers are used widely in breeding for fungal disease resistance in grain lupins [86,87,88,89,90,91], this is not yet the case with lupin virus disease resistances. This situation needs to be rectified so that these molecular approaches can be deployed to assist in breeding grain lupin cultivars with resistance to BYMV and CMV.

Genetic engineering studies examining whether viral gene constructs could introduce BYMV and CMV resistance into narrow-leafed and yellow lupins proved disappointing because these constructs were unstable in subsequent generations (see Section 2.1 and Section 2.2 above). Nevertheless, newer approaches towards genetic modification for the introduction of virus resistance, such as RNA silencing and genome editing [92,93,94], still hold considerable promise as means of introducing stable virus resistance into lupins. If they prove effective, future studies involving genetic manipulation for virus resistance should focus on combining these newer procedures with speed breeding [95,96,97] to help accelerate the introduction of BYMV and CMV resistance into grain lupins.

## 3. Conclusions

This review describes how optimized field screening procedures for virus resistance involving the introduction of virus inoculum using virus-infected transplants or spreader rows sown with virus-infected seed are used to identify potentially virus-resistant lupin germplasm accessions, breeding lines, and cultivars. It also describes how virus inoculation in the glasshouse is used to confirm suspected resistance in plants of different genotypes, to characterize resistance, and to establish its inheritance in progeny lupin plants obtained from crosses between virus-resistant and susceptible parental plants. All grain lupin species become infected with BYMV. Yellow, narrow-leafed, and pearl lupins are CMV and AMV hosts, as is white lupin for AMV, but sandplain lupin is a non-host of CMV and AMV. Several different kinds of virus resistances occur amongst different genotypes of yellow, narrow-leafed, white, and pearl lupins. Such resistances provide a key enabler towards fast tracking gains in grain lupin breeding. 

Extreme resistance to CMV was found in pearl lupin genotype P26956, but not in yellow lupin, narrow-leafed lupin, or *L. hispanicus*. Extreme resistance is normally non-strain-specific and controlled by single genes, so P26956 is likely to be useful for breeding CMV-resistant pearl lupin cultivars. Useful partial resistance to aphid transmission of BYMV was found in yellow and white lupin, and it is suspected, but not yet confirmed, for both early (i.e., systemic death symptoms) and late (i.e., BPS) infection with necrotic strain BYMV in narrow-leafed lupin. Useful partial resistance to CMV seed transmission occurs in both yellow and narrow-leafed lupin, and to BYMV seed transmission in yellow lupin. Both types of partial resistance are being used to breed for BYMV resistance in European white and yellow lupin breeding programs and for CMV seed transmission resistance in the Australian narrow-leafed lupin breeding program. In Australian studies, strain-specific LHR to CMV was found in some genotypes of yellow and pearl lupin and of *L. hispanicus*, and two genotypes of pearl lupin developed LHR when inoculated with a single AMV isolate. The LHR to CMV in yellow lupin had broad strain specificity and was controlled by the single resistance gene *Ncm-1*. One of two CMV strain-specific LHR phenotypes found in *L. hispanicus* genotypes behaved similarly, but the other LHR phenotype had narrow strain specificity. In European studies, LHR developed in white lupin inoculated with AMV. In Australian studies, LHR developed when pearl lupin was inoculated with a single AMV isolate. Use of CMV resistance gene *Ncm-1* was recommended for use in yellow lupin breeding. The LHR to AMV found in white and pearl lupin requires further study. The SHR that developed when BYMV’s necrotic strain infected narrow-leafed lupin plants was absent when they were infected by its non-necrotic strain. The presence of two strain-specific, independently inherited genes that control SHR was suggested when a diverse range of necrotic strain isolates were inoculated to plants of cultivars Danja and Merrit and of genotypes 90L423-07-13 and P26697. The SHR gene present in the two cultivars but not in 90L423-07-13 and P26697 was identified and named *Nbm-1*, but the existence of the putative SHR gene present in 90L423-07-13 and P26697 still requires confirmation. The inadvertent selection of new cultivars that behave like 90L423-07-13 and P26697 should be avoided in narrow-leafed lupin breeding, and a search is still needed for resistance to the non-necrotic BYMV strain. Field experiments in which both BYMV strains were spreading in plots of narrow-leafed lupin demonstrated the benefits of SHR controlled by resistance gene *Nbm-1* despite the rapid death of infected plants. This was because necrotic strain-infected plants were soon killed, removing them as sources for further virus spread by aphid vectors and therefore resulting in greater virus spread and grain yield losses caused by the non-necrotic strain than by the necrotic strain. When both BYMV strains spread from subterranean clover pastures into narrow-leafed lupin crops, the greater initial incidence of the necrotic strain is due to its greater incidence in the source pastures from which both strains are arriving. Regarding BPS development following late infection of narrow-leafed lupin with necrotic BYMV, more research is needed to determine if the slow BPS development trait found in cv. Jenabillup could be used to help breed new cultivars for BPS resistance. 

Finally, despite the finding that initially promising BYMV and CMV gene constructs incorporated into transgenic yellow and narrow-leafed lupins became unstable after several generations and, therefore, could not be taken further, it would still be worthwhile to explore the potential of newer genetic modification procedures. This would involve establishing whether using genome editing and RNA silencing can succeed in incorporating stable BYMV and CMV resistance into grain lupin species. Speed breeding would accelerate the release of new cultivars with such virus resistances.

## Figures and Tables

**Table 1:** Viral pathogens causing diseases in grain lupin species.

Pathogen	Virus Genus	Mode of Vector Transmission	Main Disease Symptoms	World Regions Where Lupin Infection Reported	Narrow-Leafed Lupin	White Lupin	Yellow Lupin	Pearl Lupin	Sandplain Lupin
Main pathogens									
Bean yellow mosaic virus *	*Potyvirus*	Aphid	Leaf mosaic, chlorosis, narrowing, deformation, plant stunting, or strain-specific systemic necrosis or black pod syndrome	Australasia, Europe, North and South America, Southern Africa	+++++	+++++	+++++	++++	+++++
Cucumber mosaic virus *	*Cucumovirus*	Aphid	Leaf mosaic, chlorosis, downcurling, plant stunting	Australasia, Europe, South America, Southern Africa	+++++	-	+++++	++++	-
Minor pathogens									
Alfalfa mosaic virus *	*Alfamovirus*	Aphid	Mild leaf mosaic, downcurling, plant stunting	Australasia, Europe	+++	(+)	++	+	-
Bean common mosaic virus	*Potyvirus*	Aphid	Mild leaf mosaic, deformation, stunting	Europe	(+)	-	+	-	-
Bidens mottle virus	*Potyvirus*	Aphid	Leaf narrowing, rugosity	North America	+	-	-	-	-
Broad bean wilt virus	*Fabavirus*	Aphid	Mosaic, leaf deformation, shoot apical necrosis, necrotic stem streaking, plant stunting, death	Europe	-	-	+	-	-
Clover yellow vein virus	*Potyvirus*	Aphid	Leaf chlorosis, necrotic spotting, shoot apical necrosis, stem necrosis, plant stunting	Australasia, Europe	++	+	+	-	-
Pea early browning virus	*Tobravirus*	Nematode	Necrotic stem streaking, shoot apical necrosis	Europe	-	(+)	+	-	-
Pea enation mosaic virus	*Enamovirus*	Aphid	Leaf deformation, axillary shoot proliferation	Europe	-	-	+	-	-
Peanut stunt virus	*Cucumovirus*	Aphid	Severe leaf and flower deformation, plant stunting	Europe	+	+	+	-	-
Lettuce necrotic yellows virus	*Cytorhabdovirus*	Aphid	Leaf chlorosis, plant stunting	Australasia	+	+	-	-	-
Tobacco streak virus	*Ilarvirus*	Thrips	Not reported	North America	(+)	(+)	-	-	-
Tomato black ring virus	*Nepovirus*	Nematode	Leaf mosaic, deformation, necrotic spotting, plant stunting	Europe	(+)	(+)	+	(+)	-
Tomato spotted wilt virus	*Orthotospovirus*	Thrips	Leaf ringspots (chlorotic or necrotic), deformation, and necrosis (stem streaking or dieback)	Australasia, Europe, North America	+	+	-	+	+
Soybean dwarf virus	*Luteovirus*	Aphid	Leaf chlorosis, reddening, and cupping/rolling	Australasia, East Asia	+	+	+	-	+

* = host resistance studies; +++++ = very important, ++++ = important, +++ = moderately important, ++ = minor importance, + unimportant; (+) = from glasshouse inoculations; - = no record found. The principal sources of the information in this table are the following published reviews and scientific papers: [7,11,18,19,20,21,22,23,24,25,26,27,28,29,30,31,32,33,34,35,36,37,38,39,40,41,42,43,44,45,46,47,48,49,50,51]; however, several other documents cited in the reference list also contributed data.

## Data Availability

Not applicable for reviews based entirely upon previously published information.
